# Experiencing Stress among Different Professional Groups in the Context of Their Age

**DOI:** 10.3390/ijerph19020622

**Published:** 2022-01-06

**Authors:** Grażyna Bartkowiak, Agnieszka Krugiełka, Paulina Kostrzewa-Demczuk, Ryszard Dachowski, Katarzyna Gałek-Bracha

**Affiliations:** 1 Faculty of Humanities and Social Sciences, Polish Naval Academy in Gdynia, ul. Śmidowicza 69, 81-127 Gdynia, Poland; g.bartkowiak@amw.gdynia.pl; 2 Faculty of Engineering Management, Poznan University of Technology, 5 M. Skłodowska-Curie Square, 60-965 Poznan, Poland; agnieszka.krugielka@put.poznan.pl; 3 Civil Engineering and Architecture Department, Kielce University of Technology, al.1000-lecia PP 7, 25-314 Kielce, Poland; tobrd@tu.kielce.pl (R.D.); galekkatarzyna93@gmail.com (K.G.-B.)

**Keywords:** coping with stress, mental health, employees of various ages, army officers, FUZZY TOPSIS method

## Abstract

The subject of this article is the identification of coping with stress and experiencing stress in three groups of the same number of people, different in terms of their occupation: professional soldiers of the Polish Army with the rank of an officer, people employed in managerial positions, and specialists working in independent positions in the context of their age. The analysis of the literature and the research carried out refer to the concept of sustainable development. This indicates the need to take care of limiting excessive stress and improving the mental well-being of all employees, regardless of the demographic characteristics and nature of the work performed. In order to identify possible differences, three types of questionnaires meeting the criteria of psychometric correctness were used (CISS, KPS, PSS-10). The obtained results were subjected to statistical analysis using the FUZZY TOPSIS method based on multi-criteria decision-making and the fuzzy logic, which was first applied in the social science. The obtained data confirmed some differentiation within the three studied groups, as well as the modifying role of age in coping and experiencing stress.

## 1. Introduction—The Idea of Sustainable Development

Diversity management, including the management of employees of various ages, fits and constitutes a significant content in the broadly understood idea of sustainability. The concept of sustainable development has been taken up in economic sciences, management sciences, and environmental sciences over the past 30 years [1,2,3,4]. Its current version has been developed on political and economic grounds. In recent years, its development has been clearly observed in many different scientific disciplines, i.e., environmental science, technical sciences, social sciences, economics, management, sociology, humanities, psychology, pedagogy, and health sciences. It is necessary to involve the whole society in the implementation of the principles of sustainable development, the need to predict and analyze the impact of decisions currently being made on the welfare of future generations, and distribution justice, emphasizing the participation of non-material aspects of quality of life in shaping welfare. This is indicated by numerous literature studies, including Jackson [5], Birau et al. [6], and van der Waal et al. [7], who recognize sustainable development as a system aimed at maximizing social benefits, including those relating to the organization and personnel and environmental policies, rather than giving priority to technological and economic growth. The formulation of this concept is based on the assumption that human behavior is simultaneously influenced by: the natural environment, social environment, and the subjectivity of an individual [8,9]. At the individual level, sustainable development concerns for the development of the individual, respect for their subjectivity, and a sense of meaning in the work performed. At the level of interpersonal relations, this includes tolerance of any difference, counteracting any discrimination, willingness to cooperate, as opposed to overly developed competition, taking into account the interests of partners, and cooperation aimed at maintaining supportive relationships based on ethical principles. At the organizational level, there are solutions and practices that lead to the implementation of two categories of goals [10,11,12]:

- resulting from the organization’s strategy, providing organizations with a relatively high level of innovation, stabilization, and at the same time, allowing employees to improve their various competences;
- increasing social and professional potential, allowing them to effectively cope with their workload.

From the macroeconomic point of view, sustainable development refers not only to leveling the differences between individual regions on a national scale (as well as Europe or the world) in the area of education, ensuring health and appropriate material conditions leading to a high quality of life. It also includes striving to preserve resources for the future generations and, to put the problem even more broadly, to maintain a high level of civilization and the possibility of further development [13]. These considerations show the need to manage diversity and promote appropriate patterns of behavior and apply good practices supporting the functioning of specific groups of employees in the workplace [14]. Unfortunately, in the literature on this subject, there is a lot of evidence indicating the perception of a greater advancement of age, combined with longer work experience of an employee, as a factor limiting the effectiveness and productivity of professional functioning, which is a synonym of a negatively treated routine at work.

The subject of this elaboration is the identification of coping with stress and experiencing stress depending on age for three professional groups (with the same number of respondents): professional soldiers of the Polish Army with the rank of officer, people employed in managerial occupations, and specialists working in independent occupations. The theoretical analysis and the research carried out refer to the concept of sustainable development, as mentioned above. There is a need to reduce excessive stress and improve the mental well-being of all employees, regardless of the demographic characteristics and the nature of the work. The authors formulate the following research problems:

1. Are there differences between the stress-coping styles preferred by officers, executives, and freelance workers and the level of stress they experience depending on their age and type of job? Which professional and age group is the most efficient and resistant to dealing with stress, and which experiences it the least?
2. Is age a moderator of the style of coping with stress among representatives of each professional groups? Is age a moderator of the style of coping with stress among representatives of each professional groups?

## 2. Age and Specificity of Stressors

The progress of civilization or the changing conditions of everyday existence mean that an increasing percentage of people live at a very fast pace, thus exposing themselves to many stressful situations. Stress is caused by many factors. Various problems that we are unable to deal with are often the direct causes of stress. Most people experience stress at work, but some people seem to be more resistant to stress than others. Why is this happening, and what is the cause of stress? In the literature can be found examples that describe that, depending on what stage of life we are at, we will experience stress differently [15,16,17]. Therefore, it can be assumed that employees with longer experience in a specific environment develop certain strategies of coping with stress, thanks to which they feel it to a lesser extent.

Considering the benefits of the age of workers, Baltes and Baltes [18] proposed a normative model for successful development throughout working life, the life-span theory of selective compensation optimization (SOC), which includes three general strategies: selection, optimization, and compensation. The “selection” category means selecting and focusing resources on those goals that seem to be the most valuable for the subject. Another category, “optimization”, relates to achieving a higher level of functioning through the development of competences (e.g., in the form of appropriate training or gaining new experience). Finally, “compensation” describes a strategy for minimizing losses by investing resources in other activities that counteract losses. While employees can use optimization and compensation as strategies for coping with stress, the same strategies can be successfully used both in the area of professional and private life in order to minimize the occurrence of stressors. This can be achieved, for example, by focusing on family goals, and consequently by focusing, for example, on work duties that involve fewer trips (e.g., by running local projects). It is obvious that not every profession or work provides such opportunities. Militarized domains tend to limit such opportunities [19]. Performing work in a militarized domain, especially for novice soldiers, requires resignation from one’s own preferences regarding the professional tasks and complete submission to the orders of the commander. Therefore, this work does not leave any unfilled space to arrange it in the own way.

However, the chosen strategy can manifest itself in different ways. For example, one particular manifestation is “*job crafting*”, which may be considered age-related. The term “*job crafting*” is understood as physical and cognitive changes that individuals make in terms of tasks or relational boundaries of their work [20,21,22]. Although, currently, in the literature on the subject, there is no conclusive research confirming the existence of this relationship, it can be expected that “*job crafting*” may be age-dependent. Older workers often attach more importance to work as a significant activity in their lives [23] and may have a wider spectrum (level of autonomy, enriching work) of the possibilities of its performance. On the other hand, older workers may experience increased levels of work-related stress [24,25,26]. The reason for such a state of affairs may be the sense of discrimination by their younger colleagues, the policy of the company’s authorities, or even rapid technological progress, which is often difficult for older people to cope with [27].

Employees with longer work experience are often perceived through the prism of various stereotypes related to their age [28,29,30], which introduce negative assumptions related to competences and their possibilities. For example, older workers are perceived as less creative [31,32], less interested in developing technology, and less suited to teamwork [33] due to greater adaptation difficulties. While the strategies contained in the theory of selective optimization with compensation Baltes and Baltes [18] may enable employees to actively reduce the occurrence of stressors in their work and private life cycle, as the opposite may also be the case, especially with regard to selection strategies. Employees may be able to engage in selection strategies as part of their current job and may not be able to do so outside their workplace. At the same time, studies have shown that older workers, compared to younger workers, are less likely to be re-employed after losing their job [30]. Consequently, switching from one company to another as part of the selection strategy may be limited for employees with long-term employment. Taken together, the different theoretical approaches create ambiguous predictions about the effects of age on stressors at work. While most age-related theories predict lower levels of stressors for older workers, age discrimination and job-change restrictions suggest the opposite. Currently, there are no known empirical studies that give a definite answer as to whether older workers deal with more, fewer, or even the same number of stressors as their younger colleagues.

### The Specificity of Stress among Professional Soldiers

One of the occupational groups most exposed to stress at work are soldiers (in particular, officer rank) [34,35]. It is influenced by discipline (much greater than in the case of civil professions), which significantly limits their freedom of action and the directive style of management. Many soldiers identify with the goals and style of their work, but not all. Often, soldiers do not agree with the imposed rules or feel an internal conflict related to the order they have to carry out, which is contrary to the norms they respect. These factors deepen job dissatisfaction and stress and cause negative emotions. Values such as honor, bravery, and courage sometimes help to suppress internal tears and enable soldiers to better cope with stress in their work [34,35].

The principle of operation of the army is based on an extensive system of positions and hierarchical order. Such a dependency, subordinate–superior, may be the result of stress at work, as it forces the individual to act in a style that may not be comfortable for him. Research also shows that direct contact with the supervisor has a great influence on the feeling of job satisfaction [36]. If the supervisor is satisfied with the work of the subordinate, they feel fulfilled and become even more involved in the tasks entrusted to them. Depending on his actions, a soldier can be rewarded or punished; these are two key elements (reward and punishment) that strengthen the prevailing disciplines.

Professional soldiers struggle with numerous stress factors in their daily work (about 40 such factors have been defined so far). The main causes of stress in professional soldiers include [37,38]: factors related to the way work is organized (work pace, excess tasks, lack of appropriate equipment, extended working time, etc.); the type and scope of the employee’s responsibilities (ambiguity of goals, difficulty in reconciling the professional role and family responsibilities); interpersonal relations (responsibility for employees, lack or insufficient support of superiors); employee’s professional career (too fast or too slow professional development, lack of development prospects, or uncertainty of the future); and team management style. These factors may vary in severity and usually do not appear singly but form a set of stress factors that hitting the employee even more.

## 3. Research Method

Understanding the specificity of the functioning of professional soldiers under stress becomes more noticeable if we compare their behavior with the behavior of other professional groups. It can be expected that the selection of representatives of different professions will make it possible to capture the diversity of mutual relations of the studied variables. Apart from the Polish Army officers, the survey was attended by representatives of the management staff and employees working in specialist, independent positions (mainly IT specialists). All professional groups were selected so that they were identical in terms of age, level of education (all respondents had higher education), and gender (there were two women in each group).

A partial version of the discussed research has already been published in the article “Experiencing stress among Polish Army officers, managers and people in specialist positions in the context of their age” [39] and in and multi-author article: “Styles of Coping with Stress as a Factor Influencing Professional Burnout among Professional Officers of the Polish Army in the Context of Their Age” [40].

The conducted considerations allowed for the formulation of the following problems and research hypotheses.

### 3.1. Research Problems and Hypotheses

Research problems:

1. Are there differences between the stress-coping styles preferred and the level of stress experienced by officers, executives, and freelance workers depending on their age and type of job? Which professional and age group is the most efficient and resistant to dealing with stress and experiencing it?
2. Is age a moderator of the style of coping with stress and experiencing it among representatives of particular professional groups?

Hypotheses:

**Hypothesis** **1.** *There are differences in the choice of the style of coping with stress and experiencing stress depending on the age and profession of respondents.*

**Hypothesis** **2.** *Age moderates the style of coping with stress and its experience among representatives of particular professional groups.*

### 3.2. Data Collection Tools

#### 3.2.1. Questionnaire for Coping with Stress—CISS

The CISS questionnaire allows to determine how the respondents cope with stress. The questionnaire gives an answer to the question of whether a given person increases the level of stress through their behavior or whether they are able to deal with it adequately. In the CISS questionnaire, it is possible to define which way of coping with stress from the four distinguished styles the respondent prefers:

- Task-focused style—SSZ;
- Emotion-focused style—SSE;
- Avoidance-focused style—SSU;
- Engaging in alternative activities (ACZ) or looking for social contacts—PKT.

The CISS questionnaire consists of 48 items. The respondent determines on a 5-point scale the frequency with which they take a given action in difficult and stressful situations. Reliability was measured by high internal consistency of individual scales (coefficients within the range of 0.78–0.90) and satisfactory stability (correlation coefficients between two tests at an interval of 2–3 weeks within the range of 0.73–0.80).

The theoretical validity was checked by looking for the relationship between the styles of coping with stress and personality traits, temperamental traits, anxiety, intelligence, social competence, and emotional intelligence. The analysis of criterion validity included, among others, the comparison of the results in the CISS of various professional and clinical groups. Among other things, the new validation data show the relationship between CISS scores and various aspects of professional functioning [41].

#### 3.2.2. Perception of Stress Questionnaire (KPS)

The questionnaire was constructed on the basis of theoretical supposition, assuming the legitimacy of studying the multidimensional structure of stressful experiences. The analysis of theoretical concepts as well as practical experiences allowed to focus on three basic areas of stress [42]:

- Experiencing emotional tension;
- Intrapsychic stress (resulting from confrontation with oneself);
- External stress (resulting from the confrontation of the individual with the burdens perceived in the social and external world).

The reliability of the KPS test was verified by the internal consistency coefficients for the three scales (dimensions) found in the studies of adults; this ranged from 0.70 to 0.81. Stability was not checked. The factor accuracy of KPS was confirmed. The theoretical validity was checked by correlating the KPS results with the results of the following questionnaires: personality (NEO-Five Factors Inventory—NEO-FFI), temperament (Pavlovian Temperament Survey—PTS), depression (Beck Depression Inventory—BDI), anxiety (State-Trait Anxiety Inventory—STAI), parental attitudes (Parental Attitudes Scale 2—SPR-2), attachment styles (Questionnaire of Attachment Styles—KSP), and marriage satisfaction (Well-Matched Marriage Questionnaire—KDM). Information on criterion validity is provided by comparing the results in KPS in hospitalized patients and healthy people, as well as in people working in different professions [42].

#### 3.2.3. The Scale of Perceived Stress (PSS-10)

The Perceived Stress Scale PSS-10 examines how people respond to stressful events that involve evaluating the events. This tool alludes to the Social Readjustment Rating Scale and Thomas Holmes and Richard Rahe’s List of Stressful Life Events [43].

Internal consistency was checked in a study of 120 adults, giving a Cronbach’s alpha index of 0.86. Correlation of all questions with the overall scale result is satisfactory. Reliability established on the basis of double testing of a group of 30 students, 2 days apart, was 0.90, and 0.72 after 4 weeks. PSS-10 positively correlates with the intensity of occupational stress at work, assessed on the basis of the Subjective Work Assessment Questionnaire. In their own research, the scale results were correlated with the results measured by the COPE scale, Rosenberg’s SES, Schwarzer GSES et al. The PSS-10 scale accurately measures subjective feelings related to personal problems and events and the ways of dealing with them [44,45].

### 3.3. Characteristics of the Respondents

Professional soldiers (the rank of an officer) from eight randomly selected military units in Poland participated in the research. The condition for conducting the research was the consent of the commander of the unit, although direct participation in the research was voluntary.

The research was carried out in two stages. Selection for research was deliberate. The first stage of the research involved participation in the research of officers from the Tactical Air Base.

The remaining participants of the study were people employed in managerial positions or entrepreneurs—owners of construction companies or companies dealing with the preparation of construction projects and architectural planning, as well as specialists working in the IT sector. These studies were conducted among 34 officers (including two women), for each professional group. The age range of the study participants oscillated around 24 and 43 years old, and the average age was about 36 years (Table 1).

The second stage of the research involved groups of the same number of people (34 persons of each group) who worked in the same place for at least 5 years. Participation in the research was preceded by a freely given consent and was attended by people training in postgraduate studies at universities. During the classes at the aforementioned postgraduate studies, the research participants were asked if they would like to participate in research on the experience of stress in particular age groups and representatives of various professions. Their informed consent was the condition for participation in the research.

The research participants were employed in small and medium-sized enterprises (in the case of business owners and representatives of management, it was 28 enterprises, and in the case of IT specialists, 34 enterprises, mainly service companies, most often from the construction sector) and in a group of specialists employed in independent positions (from 34 companies dealing with design work and providing IT services). There were two women in each of the three study groups.

### 3.4. Ethic Research

Participation in the study of Polish Army officers was preceded by obtaining the consent of the Ministry of National Defense, and then the consent of the commander of the military unit, which, in turn, was issued if all members voluntarily consented to participate in the research. The study was conducted in accordance with research ethics. The research problems posed took into account ethical problems of social and individual nature, i.e., with regard to the content of their formation and presentation to respondents. In the conducted study, the respondents were informed about the specific research procedures undertaken, the tools used, the objectives of the study, and what the obtained data will be used for. All participants knew that they could resign from taking part in the study at any time. Each of the respondents was guaranteed anonymity. The conducted research did not in any way compromise the sense of freedom and the moral values system of each of the respondents [46].

### 3.5. FUZZY TOPSIS Method—Multi Criteria Decision Making

In order to obtain answers to the questions of research problems and to verify research hypotheses, the multi-criteria decision-making method was used. Multi-criteria decision-making as a research method has found application in fields investigating complex research problems. Among the methods of multi-criteria decision-making, a group of methods using fuzzy numbers can be distinguished. The fuzzy approach is used in order to reduce the subjectivity of the performed assessment. The FUZZY TOPSIS method has been used, among other disciplines and studies, in areas such as construction [47] and logistics.

C.J. Park et al. used the FUZZY TOPSIS method to examine the factors responsible for employee retention in the construction sector [48] (Rank Determinants of Employee Retention in Construction Companies), while H. Jaber et al. investigated the A Framework to Evaluate Project Complexity [49]. Jellali et al. [50] carried out a sustainable configuration of the Tunisian olive oil supply chain using a FUZZY TOPSIS-based approach (Sustainable Configuration of the Tunisian Olive Oil Supply Chain Using a FUZZY TOPSIS-Based Approach). Supply issues were also a struggle in A. Pinar et al. [51].

The article uses the FUZZY TOPSIS approach proposed by Chen [52]. Fuzzy logic (and fuzzy numbers) are used when studies are not entirely sure whether the obtained data, for example, from surveys, are actually correct, i.e., whether the respondents are 100% sure that they have given the correct answer. This is known as the uncertainty (fuzzy) environment. Another advantage of this method in the case of studying the phenomenon of stress, compared to other methods, is a comprehensive approach to the behavior of the studied groups in the face of a difficult situation (coping with stress and experiencing stress), taking into account the results and all three questionnaires. The method determines the ideal solution and the opposite to the ideal solution. The distances of variants from ideal and anti-ideal solutions are calculated, which shows the relative proximity to the ideal and anti-ideal solutions [53]. The description of the decision problem is presented using a decision matrix containing criteria and decision variants. In the analysis presented in this paper, the assessments of decision makers were organized by grouping the respondents according to age ranges. Ratings are presented using fuzzy numbers. The analysis considered criteria belonging to two groups. For the criteria from the “profit” group, the highest value was preferred, while for the criteria from the “cost” group, the lowest values were desirable. The decision regarding the classification of individual statements to the above-mentioned criteria was made on the basis of the method of competent judges, who were experts from both social and technical sciences. The assignment was made with reference to the theories underlying the construction of research tools. The next steps of the method consist of data normalization and assigning weight vectors to them. The result of the performed calculations is obtaining a ranking of decision variants. For the purposes of this study, it was examined which of the groups (officers, managers, or specialists) experience the most stress, and which are better able to deal with it and experience less stress and to a lower extent.

The inspiration for the choice of the FUZZY TOPSIS method was the repeated observations of researchers, which allowed them to notice that, in the surveys conducted, people participating in the study often have problems with giving clearly formulated, unambiguous answers. Introducing ambiguity in the response leads to a situation in which a choice must be made among at least two alternatives [49]. Therefore, fuzzy logic was introduced, combining numerical and symbolic modeling. Fuzzy logic converts numerical values into linguistic terms. The sets used in fuzzy logic are described by the membership function. The membership function determines to what part an element belonging to the set is included in the fuzzy set. The membership of the functions is determined with values from 0 to 1. Language terms have been converted to fuzzy numbers based on a scale containing values from 1 to 9 (Table 2).

### 3.6. Application of the FUZZY TOPSIS Method to Determine Stress in the Studied Groups

The FUZZY TOPSIS method was used to investigate differences in coping styles and experiencing stress in the context of the moderating role of age among professional Polish Army officers, representatives of management staff, and specialists employed in independent positions. Information from previously conducted questionnaires was used as criteria in the analysis. The decision-making variants in the analysis were representative of the studied groups. The participants are divided into age groups. In the conducted analysis, due to the uniformity of the criteria used, uniform weights were adopted for them.

The decision to apply specific criteria for assigning variables was made on the basis of the adopted theory of stress, indicating the mechanism of the impact of stressors on human behavior. Research using the CISS questionnaire has been repeatedly carried out throughout Poland [54], treating the choice of a task-based stress coping strategy (SSZ) as the optimal style of overcoming mental stress. The presented style is chosen by people who, in stressful situations, show a tendency to make efforts to solve problems through cognitive transformations or attempts to change the situation. This style can be explained by referring also to the salutogenic model of stress. This model assumes that, in many cases, man has no influence on the reduction in or complete elimination of stress, but he should shape his external world in such a way that he can independently function and develop creatively in it [55]. According to this approach, stressors are a natural phenomenon, and creative adaptation consists of acquiring individual experience, including experiencing stress over and over again, by perceiving the world as controllable, meaningful, and understandable. Such an emotional–cognitive orientation towards the world is composed of three components: comprehensibility, manageability, and meaningfulness [55,56]. This style is dominated by the emphasis placed on the task or problem-solving planning. In the KPS test (emotional tension, external stress of intrapsychic, adjustment overall), a high KPS stress score indicates low resilience, regardless of whether it is a personality trait or a process (cf. normalization of the KPS test results—Plopa and Makarowski research) [42]. Stress measured using the Perceived Stress Scale (PSS-10) is used to assess the stress related to one’s own professional situation over the last month. The combination of high levels of stress and its long duration leads to more serious effects of stress, including health effects [57].

## 4. Findings

### 4.1. FUZZY TOPSIS Method

Fuzzy logic (and fuzzy numbers) are used when research members are not entirely sure whether the data obtained from the questionnaires are actually correct, i.e., whether the participants are 100% sure that they have given the correct answer, which is referred to as the uncertainty environment. The decision variants and criteria after normalization and taking into account the weights are presented in Table 3.

### 4.2. Differences in Terms of the Level of Experiencing Stress in Individual Professional Groups of People Participating in the Research

According to the presented analysis, after carrying out the calculations appropriate for the FUZZY TOPSIS method, the following ranking was obtained (Table 4 and Table 5).

The obtained data made it possible to observe differences in the styles of coping and experiencing intrapsychic stress between the group of officers, representatives of management, and people employed in independent positions. Thus, while the differences are minor, the officers as a group experience somewhat less stress than the specialists and then the management. This may mean that they have a more consolidated system of values, a higher level of sense of the work they perform, and at the same time, a different type of responsibility, which (except in special cases) does not result in resignation from the workplace in the event of failure. Moreover, officers can be expected to use specific strategies to reduce the experience of stress.

The obtained data confirmed the first and the second adopted hypothesis. It turns out that there are individual differences in the style of coping with stress and the level of experiencing stress between all the studied groups. In addition, it was possible to confirm that the oldest people (especially in the group of specialists and managers) with greater work experience cope with stress better (e.g., by choosing the right strategy) and experience less stress than in the case of younger colleagues.

## 5. Summary

### 5.1. Discussion of the Results

The obtained research results confirmed the hypothesis about the existence of differences in the level of experiencing stress in individual professional groups of people participating in the research in the context of age and the hypothesis about greater ability to cope with stress and experience stress among older research participants. This state could be caused by both personality conditions (in the case of the group of Polish Army officers), revealed in the recruitment and selection procedure, as well as professional experience (that made it possible to learn about a wide repertoire of individually available strategies limiting the experience of stress, and then to make a specific choice). The lower level of experience of stress in the group of officers, compared to representatives of management and independent employees, may also result from the beneficial effect of the organized training courses.

As people with longer professional experience and a certain autonomy at work, they may have a greater opportunity to shape their work (job crafting) both in the structural aspect, i.e., some modification of professional sentences and the way they are performed, as well as relational by building cooperation in teams, in such a way that they are performed by the person best prepared for it. Therefore, due to their professional experience, they may apply a selective optimization strategy with compensation. This strategy, similarly to job crafting, boils down to a certain selection and optimization of performed tasks, adapting them to personal competences and preferences, and if possible, compensating, i.e., replacing one activity with another. On the other hand, controversial is the fact that there is a discrepancy in the field of coping and experiencing stress with the highest rank of specialists, compared to representatives of the management staff who, due to their positions, seem to maintain greater autonomy at work, enabling the use of strategies, and professional officers of the Polish Armed Forces.

However, there are few studies focused on promoting the breaking of stereotypes, an interdisciplinary approach, and from the point of view of the social economy, and from the point of view of broadly understood environmental psychology. Some researchers emphasize that there is a significant necessity to improving the psycho-social working conditions as workers age by combating ageism in the workplace [28,33]. The conducted research fills this gap.

Analysis of the research results confirms the assumptions of other researchers [15,16,17], which “spoke out” about the relationship between age and stage of life on experiencing stress at work.

Additionally, according to other researchers, self-efficacy and the use of positive coping strategies that reduce the experience of stress may help reduce stress levels, but they cause lower burnout [58,59,60].

Referring the above findings to the conducted research, which showed a lower level of experienced stress in the group of the surveyed officers of the Polish Armed Forces with a longer service, some assumptions can be made. For example, with increase in seniority and professional experience, officers were more willing to apply certain primary and secondary strategies (or both at the same time) as an instrument of reducing the experience of stress and, consequently, possibly of lower occupational burnout. In their case, although they work in a militarized domain, it would be due to modifying working conditions, selecting their own activity as much as possible, and optimizing work (by acquiring new competences during training and gaining experience, recognizing own strengths) and the result of undertaking of activities that are to some extent autonomous but, at the same time, compensatory. Such an interpretation is also a confirmation of the strategy of coping with stress within the framework of the theory of selection and optimization with compensation proposed by the Bales [18] as well as shaping work [20,21,22,61] and the theory of primary control.

### 5.2. Research Limitation

The presented studies are not free from limitations. One of them is the size of the research sample, numbering a total of 102 people, as well as not very diverse age diversification of the respondents. Due to this situation, the observed age discrepancy was, on average, around 20 years (the youngest person was 23 years old, and the oldest person was 48 years old). For this reason, in the opinion of the authors of the article, it would be good to repeat the research paradigm on a larger research group, more diverse in terms of age and professional groups. Furthermore, recognizing the preferences of the applied strategy of reducing the experience of stress would require planning qualitative research, allowing for the identification of factors determining the selection of a specific strategy within individual occupational groups, in particular, groups with a higher risk of experiencing stress. When considering how to optimize future research, it would be a good idea to include a seniority of service with the same organization as another variable. In the presented research, only a minimum of 5 years of experience in the same workplace was adopted as the criterion for selecting people for the research. However, subsequent studies could diversify the studied groups of people depending on their seniority in one workplace and then compile and compare the obtained data.

## 6. Conclusions

The presented research results, apart from the cognitive value, which include the development of knowledge about the relationship of the studied variables—coping with and experiencing stress in the context of age and belonging to a specific professional group, allow for the formulation of the following application conclusions at the level of individual organizations and the system:

- There is a need for research and continuous verification of the level of experiencing stress in various professional and age groups, in particular in professions with an increased risk of stress;
- The research should lead to the recognition of subjective causes of stress in individual employees and entire professional groups;
- It would be advisable to organize trainings increasing the level of individual resources conditioning the experience of stress (professional, social competences and resilience in a stressful situation) in individual age groups, allowing individuals to limit the experience of stress in accordance with the resented earlier strategies (strategy of selection and optimization with compensation, job shaping, crafting), primary external; secondary, internal;
- One should strive to identify organizational sources of stress and undertake specific organizational intervention;
- It would be good idea to promote the professional achievements of people with longer professional experience in order to counteract a possible, often collapsed form of discrimination against older workers;
- Considering the personality conditions of experiencing stress, in professions with an increased risk of stress, particular attention should be paid to the recruitment and selection procedure.

Thus, the results obtained with the use of the FUZZY TOPSIS method based on the logic of fuzzy numbers (negating the existence of ideal and anti-ideal solutions) allowing for a more objective approach to the obtained research results [52,53] showed that, among the studied groups, age is a significant criterion differentiating the choice of coping style and the experience of stress. The obtained results confirm the possibility of using the FUZZY TOPSIS method in social sciences, which, apart from the aforementioned advantages, e.g., compared to regression analysis, seems to be a much more economic method. Moreover, in the case of the research, the above-mentioned and simultaneously applied multi-criteria approach is particularly valuable, allowing for the ranking of the surveyed groups of people.

## Figures and Tables

**Table 1:** Age of the respondents—descriptive statistic. Source: own research.

Data	Officers	Managers	Specialists (Independent Workers)
Average	36.097	36.106	35.872
Median	36	35	33
Dominant	29	30	28
Standard deviation	5.556	6.221	5.002
Minimum	24	25	23
Maximum	43	48	41

**Table 2:** Fuzzy score and linguistic terms. Source: own research.

Fuzzy Score	Linguistic Terms
(1, 1, 3)	Very low
(1, 3, 5)	Low
(3, 5, 7)	Medium
(5, 7, 9)	High
(7, 9, 9)	Very high

**Table 3:** Criteria and decision options. Source: own research.

Decision Options	Criteria									
	SSZ	SSE	SSU	ACZ	PKT	Emotional Tension	External Stress	Stress of Intraps.	Adjusted Overall	PSS
group 1 section 24–30	0.06	0.01	0.01	0.01	0.01	0.01	0.01	0.01	0.01	0.01
	0.08	0.01	0.02	0.02	0.01	0.01	0.01	0.01	0.01	0.01
	0.1	0.02	0.03	0.03	0.02	0.01	0.01	0.01	0.02	0.02
group 1 section 31–35	0.01	0.01	0.02	0.01	0.01	0.02	0.01	0.03	0.02	0.01
	0.03	0.01	0.03	0.02	0.01	0.03	0.02	0.02	0.03	0.01
	0.06	0.02	0.1	0.03	0.02	0.1	0.03	0.03	0.1	0.01
group 1 section 36–40	0.03	0.02	0.03	0.03	0.01	0.01	0.01	0.03	0.02	0.01
	0.06	0.03	0.1	0.1	0.02	0.02	0.02	0.1	0.03	0.01
	0.08	0.1	0.1	0.1	0.03	0.03	0.03	0.03	0.1	0.01
group 1 section 41–45	0.08	0.02	0.01	0.02	0.01	0.02	0.01	0.03	0.02	0.01
	0.1	0.03	0.02	0.03	0.01	0.03	0.02	0.03	0.03	0.01
	0.1	0.1	0.03	0.1	0.02	0.1	0.03	0.03	0.1	0.02
group 1 section 46–50	0.06	0.02	0.01	0.02	0.01	0.02	0.02	0.1	0.01	0.01
	0.08	0.03	0.01	0.03	0.01	0.03	0.03	0.02	0.02	0.01
	0.1	0.1	0.02	0.1	0.01	0.1	0.1	0.1	0.03	0.01
	**SSZ**	**SSE**	**SSU**	**ACZ**	**PKT**	**Emotional Tension**	**External Stress**	**Stress of Intraps.**	**Adjusted Overall**	**PSS**
group 2 section 24–30	0.03	0.01	0.01	0.01	0.01	0.01	0.01	0.01	0.01	0.01
	0.06	0.01	0.01	0.01	0.01	0.01	0.01	0.01	0.01	0.02
	0.08	0.01	0.01	0.01	0.01	0.01	0.01	0.01	0.01	0.03
group 2 section 31–35	0.01	0.01	0.01	0.01	0.01	0.01	0.02	0.1	0.01	0.01
	0.01	0.01	0.01	0.01	0.01	0.02	0.03	0.01	0.01	0.01
	0.03	0.01	0.02	0.02	0.01	0.03	0.1	0.1	0.02	0.02
group 2 section 36–40	0.01	0.01	0.01	0.01	0.01	0.01	0.01	0.03	0.01	0.02
	0.03	0.01	0.02	0.01	0.02	0.02	0.02	0.02	0.02	0.03
	0.06	0.02	0.03	0.02	0.03	0.03	0.03	0.03	0.03	0.1
group 2 section 41–45	0.06	0.01	0.02	0.02	0.01	0.03	0.03	0.1	0.03	0.02
	0.08	0.02	0.03	0.03	0.01	0.1	0.1	0.03	0.1	0.03
	0.1	0.03	0.1	0.1	0.02	0.1	0.1	0.1	0.1	0.1
group 2 section 46–50	0.01	0.03	0.03	0.03	0.03	0.03	0.03	0.1	0.03	0.03
	0.01	0.1	0.1	0.1	0.1	0.1	0.1	0.1	0.1	0.1
	0.03	0.1	0.1	0.1	0.1	0.1	0.1	0.1	0.1	0.1
	**SSZ**	**SSE**	**SSU**	**ACZ**	**PKT**	**Emotional Tension**	**External Stress**	**Stress of Intraps.**	**Adjusted Overall**	**PSS**
group 3 section 24–30	0.01	0.01	0.01	0.01	0.03	0.01	0.01	0.02	0.01	0.01
	0.03	0.01	0.01	0.01	0.1	0.01	0.01	0.01	0.01	0.01
	0.06	0.02	0.01	0.01	0.1	0.02	0.02	0.02	0.01	0.02
group 3 section 31–35	0.01	0.01	0.01	0.01	0.02	0.01	0.01	0.01	0.01	0.02
	0.03	0.01	0.01	0.01	0.03	0.01	0.01	0.01	0.01	0.03
	0.06	0.01	0.01	0.01	0.1	0.02	0.01	0.01	0.01	0.1
group 3 section 36–40	0.03	0.01	0.01	0.01	0.02	0.01	0.01	0.02	0.01	0.01
	0.06	0.02	0.01	0.01	0.03	0.01	0.01	0.01	0.01	0.02
	0.08	0.03	0.02	0.02	0.1	0.01	0.02	0.02	0.01	0.03
group 3 section 41–45	0.06	0.02	0.01	0.01	0.02	0.01	0.01	0.02	0.01	0.03
	0.08	0.03	0.02	0.02	0.03	0.01	0.01	0.02	0.02	0.1
	0.1	0.1	0.03	0.03	0.1	0.02	0.02	0.02	0.03	0.1
group 3 section 46–50	0.08	0.03	0.03	0.02	0.02	0.01	0.03	0.1	0.03	0.03
	0.1	0.1	0.1	0.03	0.03	0.02	0.1	0.1	0.1	0.1
	0.1	0.1	0.1	0.1	0.1	0.03	0.1	0.1	0.1	0.1

**Table 4:** FUZZY TOPSIS ranking. Source: own research.

Decision Options	dj+	dj−	Sj	Rank Position
group 1 section 24–30	0.210	0.033	0.327	13
group 1 section 31–35	0.179	0.160	0.464	8
group 1 section 36–40	0.150	0.239	0.615	4
group 1 section 41–45	0.166	0.184	0.568	6
group 1 section 46–50	0.165	0.217	0.589	5
group 2 section 24–30	0.219	0.021	0.233	14
group 2 section 31–35	0.193	0.152	0.424	9
group 2 section 36–40	0.187	0.105	0.359	12
group 2 section 41–45	0.130	0.294	0.666	3
group 2 section 46–50	0.084	0.385	0.733	2
group 3 section 24–30	0.193	0.126	0.408	10
group 3 section 31–35	0.203	0.126	0.380	11
group 3 section 36–40	0.201	0.095	0.359	12
group 3 section 41–45	0.174	0.182	0.542	7
group 3 section 46–50	0.083	0.342	0.814	1

**Table 5:** Summary of results for individual groups. Source: own research.

Study Group	The Sum of Sj for All Ranges	Medium Sj
group 1	2.56	0.51
group 2	2.41	0.48
group 3	2.50	0.50

## Data Availability

The data presented in this study are available on request from the corresponding author. The data are not publicly available due to restrictions privacy.

## References

[1] Staric M., Marcus A.A. (2000). Introduction to the special research forum on the management of organizations in the natural environment. Acad. Manag. J..

[2] Etzion D. (2007). Research on organizations and the natural environment, 1992-present: A review. J. Manag..

[3] Buffa F., Frach M., Rizo D. (2018). Environmental management practices for sustainable business models in small and medium hotel enterprises. J. Clean. Prod..

[4] Gladwin T.N., Kennelly J.J., Krause T.S. (1995). Shifting paradigms for sustainable development. Acad. Manag. Rev..

[5] Jackson T. (2009). Prosperity without Growth: Economics for a Finite Planet.

[6] Birau F.R., Dănăcică D.E., Spulbar C.M. (2019). Social exclusion and labor market integration of people with disabilities, a case study for romania. Sustainability.

[7] Van der Waal J.W.H., Thijssens T., Maas K. (2021). The innovative contribution of multinational enterprises to the sustainable development goals. J. Cleaner. Prod..

[8] GhaffarianHoseini A., Tookey J., GhaffarianHoseini A., Naismith N., Olabode Bamidele Rotimi J. (2016). Integrating alternative technologies to improve built environment sustainability in Africa: Nexus of energy and water. Smart Sustain. Built Environ..

[9] Chin T., Li G., Hao J., Addo F., Jawahar I.M. (2019). Career sustainability during manufacturing innovation A review, a conceptual framework and future research agenda. Career Dev. Int..

[10] Grecu V., Ciobotea R.-I.-G., Florea A. (2020). Software application for organizational sustainability performance assessment. Sustainability.

[11] Ullah Z., Álvarez-Otero S., Sulaiman M.A.B.A., Sial M.S., Ahmad N., Scholz M., Omhand K. (2021). Achieving organizational social sustainability through electronic performance appraisal systems: The moderating influence of transformational leadership. Sustainability.

[12] Westerman J.W. (2021). A sustainable plan to rescue hr from itself. Sustainability.

[13] Daigle C., Vasseur L. (2019). Is It Time to Shift Our Environmental Thinking? A Perspective on Barriers and Opportunities to Change. Sustainability.

[14] Marnewick C., Silvius G., Schipper R. (2019). Exploring Patterns of Sustainability Stimuli of Project Managers. Sustainability.

[15] Burr H., Pohrt A., Rugulies R., Holtermann A., Hasselhorn H.M. (2017). Does age modify the association between physical work demands and deterioration of self-rated general health?. Scand. J. Work. Environ. Health.

[16] Donders N.C., Bos J.T., van der Velden K., van der Gulden J.W.J. (2012). Age differences in the associations between sick leave and aspects of health, psychosocial workload and family life: A cross-sectional study. BMJ Open.

[17] Shultz K.S. (2010). Age differences in the demand-control model of work stress: An examination of data from 15 European countries. J. Appl. Gerontol..

[18] Baltes P.B., Baltes M.M., Baltes P.B., Baltes M.M. (1991). Psychological perspectives on successful aging: The model of selective optimization with compensation. Successful Aging: Perspectives from the Behavioral Sciences.

[19] Kaushal S.L., Jalan S. (2020). Occupational Stress in Himachal Pradesh Police Constabulary. J. Manag. Public Policy.

[20] Berg J.M., Dutton J.E., Wrześniewski A., Dick B.J., Byrne Z.S., Steger M.F. (2013). Job crafting and meaningful work. Purpose and Meaning in the Workplace.

[21] Bartkowiak G., Krugiełka A. (2018). Job crafting among Polish entrepreneurs and representatives of the management staff. Manag. Financ..

[22] Bartkowiak G. (2021). Job Crafting. Shaping the Subjectivity of Work—The Perspective of Work Pedagogy and Organizational Psychology.

[23] Carstensen L.L. (2006). The influence of a sense of time on human development. Science.

[24] Snyder M., Miene P.K. (1994). Stereotyping of elderly: A functional approach. Br. J. Soc. Psychol..

[25] Staudinger U.M., Bowen C.E. (2011). A synthetic approach to aging in the work context. J. Labour Mark. Res..

[26] Settersten R.A. (1998). Time, age, and transition to retirement: New evidence on life course flexibility. Int. J. Aging Hum. Dev..

[27] Huk T. (2014). Media Pedagogy. Social, Cultural and Educational Aspects.

[28] Krugiełka A. (2019). Modeling CSR in Relation to the Internal Client.

[29] Hess T., Birren J.F., Schaie W.K., Ables P.R., Gatz M., Salhouse T.A. (2006). Attitudes toward Aging and Their Effect on Behavior. Psychology of Aging.

[30] Bartkowiak G. (2016). Employing Knowledge Workers 65 Plus. Perspective of Employees and Organizations.

[31] Levin W.C. (1998). Age stereotyping: College student evaluations. Res. Aging.

[32] Pasupti M., Lockenhoff C.E., Nelson T.D. (2002). Ageist behavior. Ageism: Stereotyping and Prejudice Against Older Person.

[33] Nwachukwu I., Nkire N., Shalaby R., Hrabok M., Vuong W., Gusnowski A., Surood S., Urichuk L., Greenshaw A.J., Agyapong V.I.O. (2020). COVID-19 Pandemic: Age-Related Differences in Measures of Stress, Anxiety and Depression in Canada. Int. J. Environ. Res. Public Health.

[34] Chandel N.K. (2013). Stress Management in Indian Army. Sansmaran Res. J..

[35] Tomaszewska I. (2003). Psychosocial Determinants of Coping with Stress in the Military Professional Staff.

[36] Piotrowski A., Maciejewski J., Liberadzki M. (2011). Psychosocial variables characterizing officers of the Prison Service considering resignation from work. Recruitment to Dispositional Groups—Sociological Analysis of the Problem.

[37] Saravanan P., Panchanatham N. (2017). Impact of Motivation, Change the Stress Level of Personnel of Indian Army—An Empirical Study. Int. J. Glob. Bus. Manag. Res..

[38] Maciejewski J., Wolska-Zogata I. (2004). The Profession of an Officer in the Polish Army in the Course of Transformation. Sociological Study.

[39] Bartkowiak G., Krugiełka A. (2021). Experiencing stress among Polish Army officers, managers and people in specialist positions in the context of their age. Contin. Educ. Adults.

[40] Bartkowiak G., Krugiełka A., Kostrzewa-Demczuk P., Dachowski R., Gałek K. (2021). Styles of Coping with Stress as a Factor Influencing Professional Burnout among Professional Officers of the Polish Army in the Context of Their Age. Sustainability.

[41] Strelau J., Jaworowska A. (2020). CISS—The Coping Survey in Stressful Situations.

[42] Plopa M., Makarowski R. (2010). Sense of Stress Questionnaire.

[43] Holmes T.H., Rahe R.H. (1967). The Social Readjustment Rating Scale. J. Psychosom. Res..

[44] Jiang J.M., Seng E.K., Zimmerman M.E., Sliwinski M., Kim M., Lipton R.B. (2017). Evaluation of the Reliability, Validity, and Predictive Validity of the Subscales of the Perceived Stress Scale in Older Adults. J. Alzheimer’s Dis..

[45] Juczyński J., Ogińska-Bulik N. (2009). PSS-10—The Perceived Stress Scale.

[46] Frankfort-Nachmiast C., Nachmiast D. (2001). Research Methods in Social Sciences.

[47] Saeli M., Micale R., Seabra M.P., Labrincha J.A., La Scalia G. (2020). Selection of Novel Geopolymeric Mortars for Sustainable Construction Applications Using Fuzzy Topsis Approach. Sustainability.

[48] Park C., Soo-yong K., Minh V.N. (2021). Fuzzy TOPSIS. Application to Rank Determinants of Employee Retention in Construction Companies: South Korean Case. Sustainabillity.

[49] Jaber H., Marle F., Vidal L.A., Sarigol I., Didiez L. (2021). A Framework to Evaluate Project Complexity Using the Fuzzy Topsis Method. Sustainability.

[50] Jellali A., Hachicha W., Aljuaid A.M. (2021). Sustainable Configuration of the Tunisian Olive Oil Supply Chain Using a Fuzzy TOPSIS-Based Approach. Sustainability.

[51] Pınar A., Rouyendegh B.D., Özdemir Y.S. (2021). Q-Rung Orthopair Fuzzy TOPSIS Method for Green Supplier Selection Problem. Sustainability.

[52] Chen C.T. (2000). Extensions of the TOPSIS for Group Decision-Making under Fuzzy Environment. Fuzzy Sets Syst..

[53] Hwang C.-L., Kwangsun Y. (1981). Multiple Attributes Decision Making.

[54] Strelau J., Jaworowska A., Wrześniewski K., Szczepaniak P. (2005). The CISS Coping Questionnaire in Stress Situations.

[55] Antonovsky A. (1987). The salutogenic perspective: Toward a new view of health and illness. Advances.

[56] Söderhamn O., Holmgren L. (2004). Testing Antonovsky’s sense of coherence (SOC) scale among Swedish physically active older people. Scand. J. Psychol..

[57] Bloch G., Aleamoni L. (2005). The Salient Stressor Impact Questionnaire (SSIQ). A measurement of intensity and chronicity of Stress. Assessment.

[58] Gam J., Kim G., Jeon Y. (2016). Influences of art therapists’ self-efficacy and stress coping strategies on burnout. Arts Psychotheaphy.

[59] Pignata S., Winefield A.H., Provis C., Boyd C.M. (2016). Awareness of stress-reduction interventions on work attitudes: The impact of tenure and staff group in Australian Universities. Front. Psychol..

[60] Wu S., Li H., Zhu W., Lin S., Chai W., Wang X. (2012). Effect of work stressors, personal strain, and coping resources on burnout in Chinese medical professionals: A structural equation model. Ind. Health.

[61] Wrzesniewski A., Dutton J.E. (2001). Crafting a job: Revisioning employees as active crafters of their work. Acad. Manag. J..

